# Impact of Surface Potential on Apatite Formation in Ti Alloys Subjected to Acid and Heat Treatments

**DOI:** 10.3390/ma10101127

**Published:** 2017-09-24

**Authors:** Seiji Yamaguchi, Hideki Hashimoto, Ryusuke Nakai, Hiroaki Takadama

**Affiliations:** Department of Biomedical Sciences, College of Life and Health Sciences, Chubu University, Kasugai 487-0027, Japan; hideki-hashimoto@isc.chubu.ac.jp (H.H.); nakai.ryusuke.2u@kyoto-u.ac.jp (R.N.); takadama@isc.chubu.ac.jp (H.T.)

**Keywords:** Ti alloy, apatite, simulated body fluid, positive charge, acid treatment

## Abstract

Titanium metal (Ti) and its alloys are widely used in orthopedic and dental fields. We have previously shown that acid and heat treatment was effective to introduce bone bonding, osteoconduction and osteoinduction on pure Ti. In the present study, acid and heat treatment with or without initial NaOH treatment was performed on typical Ti-based alloys used in orthopedic and dental fields. Dynamic movements of alloying elements were developed, which depended on the kind of treatment and type of alloy. It was found that the simple acid and heat treatment enriched/remained the alloying elements on Ti–6Al–4V, Ti–15Mo–5Zr–3Al and Ti–15Zr–4Nb–4Ta, resulting in neutral surface charges. Thus, the treated alloys did not form apatite in a simulated body fluid (SBF) within 3 days. In contrast, when the alloys were subjected to a NaOH treatment prior to an acid and heat treatment, alloying elements were selectively removed from the alloy surfaces. As a result, the treated alloys became positively charged, and formed apatite in SBF within 3 days. Thus, the treated alloys would be useful in orthopedic and dental fields since they form apatite even in a living body and bond to bone.

## 1. Introduction

Titanium metal (Ti) and its alloys are widely used in orthopedic and dental fields due to their high degree of mechanical strength and good biocompatibility. However, stable fixation between the metals and bone is hardly achieved because of the absence of the bone-bonding capacities in the metals [1,2]. After the finding that Ti with a roughened surface can be in direct contact with living bone [2], grid blasting and acid etching were usually used on Ti and its alloy implants in orthopedic and dental fields [3]. However, this direct contact itself does not bond the implant to bone. Various kinds of acid such as H_2_SO_4_/HCl, H_2_O_2_/HCl, HF/HNO_3_ and H_2_O_2_/TaCl_2_ were used in the acid etching [4,5,6,7] and their effects on apatite formation in a simulated body fluid (SBF) with ion concentrations nearly equal to those of human blood plasma [8] were investigated to speculate the degree of bone-bonding capacities of the acid-treated Ti. The induction periods of apatite formation on the acid treated Ti were longer than 7 days in many cases [4,5,6], whereas they decreased to be shorter than 3 days when Ti was subsequently heat treated at moderate temperature or soaked in nitric acid solution for a long period of time [6,7]. Surface roughness, crystalline phases of anatase and rutile, amount of hydroxyl groups, surface potential and so forth have been thought to be the important factors for apatite formation on Ti [4,5,6,7,9,10]. Among them, recent research reported that surface potential is a dominant factor of apatite formation in pure Ti [4,9]. However, studies on acid- and heat-treated Ti alloys are scarce, and the impact of surface potential on apatite formation in Ti alloys subjected to acid and heat treatments remains unclear. It is reported that Ti–6Al–4V alloy subjected to H_2_O_2_ treatment forms some amount of apatite after being soaked in SBF for 15 days, but its apatite formation was not increased even after the heat treatment up to 400 °C [11]. Cell study on acid-treated pure Ti revealed that the surface with micro-scale roughness is effective in promoting osteoblast differentiation, while it depresses osteoblast proliferation compared with a smooth surface [12,13,14,15]. Zhao et al. reported that the micro-scale roughness produced on pure Ti by the HF treatment increased protein adsorption, initial cell attachment and osteogenic related gene expression, while it significantly suppressed proliferation extra cellar matrix deposition, and mineralization of primary rat calvarial osteoblast [12]. The increase of proliferation was observed on the hybrid micro/nano-textured surfaces formed on pure Ti produced by anodic oxidation following the acid treatment [12]. The increase of proliferation was also reported on micro-scale roughness of Ti–6Al–4V alloy that was thermally treated at 400 °C following the mixed acid treatment of HCl/H_2_SO_4_ [16].

On the other hand, it has been demonstrated that a bioactive nano-porous surface layer composed of sodium titanate and rutile was produced on Ti by soaking in NaOH solution at 60 °C for 24 h and then heating at 600 °C for 1 h [17]. Later, the modified alkali and heat treatments—NaOH-CaCl_2_-heat-water that forms calcium titanate on the surfaces of the Ti—were developed [18]. Both treatments conferred Ti and conventional Ti alloys such as Ti–6Al–4V, Ti–6Al–2Nb–1Ta, Ti–15Mo–5Zr–3Al high capacities for bone bonding as well as apatite formation [19,20,21], and the latter modified treatment was effective even for new types of Ti alloys such as Ti–15Zr–4Nb–4Ta, Ti–29Nb–13Ta–4.6Zr, and Ti–36Nb–2Ta–3Zr–0.3O free from elements suspected of cytotoxicity [22,23,24,25]. It was reported that pure Ti with the sodium titanate layer formed by the NaOH and heat treatment promoted osteoblast differentiation through the production of osteocalcin and significant up-regulation of Runx2, Osx, Dlx5, ALP, BSP, OC and DMP1 mRNA levels without suppression of proliferation compared with abraded Ti [26]. Furthermore, direct bone bonding of the treated Ti that led to the formation of a bone-like apatite layer on its surface in SBF was observed in the bone explant model that demonstrated migration of cells from the explants and subsequent differentiation forming a mineralized nodular structure [27]. However, the produced sodium titanate or calcium titanate by these types of alkali and heat treatments release Na^+^ or Ca^2+^ ions in the body environment. These alkali ions might give an adverse effect on living cells, when they accumulate in a narrow space of a porous body.

It was shown that when Ti was soaked in water or HCl solution after the NaOH treatment, and then subjected to the heat treatment, the surface layer free from Na^+^ ions that was composed of anatase and rutile was formed on the surface of the metal [28,29,30]. The treated metal formed apatite in SBF within 1 day and bonded to living bone, again [28,30]. When the NaOH-HCl-heat treatment was applied to porous Ti containing different numbers of interconnected pores of various sizes, newly grown bone deeply penetrated into the pores in rabbit femur as a result of osteoconduction [31]. Not only osteoconduction, but also osteoinduction—ectopic bone formation in muscle—was observed when porous Ti subjected to the same treatment was implanted into the dorsal muscle of a beagle dog [32]. This was in contrast with the porous Ti with sodium titanate or calcium titanate that exhibited only slight or no osteoinduction as a result of the adverse effect of the released Na^+^ and Ca^2+^ ions on the activity of living cells [32].

It was reported that selective removal of alloying elements was observed on the surfaces of Ti–6Al–4V and Ti–15Zr–4Nb–4Ta alloys after the NaOH treatment [17,33]. However, there are no systematical studies of the acid and heat treatment with or without initial NaOH treatment on Ti-based alloys in terms of surface potential.

In the present study, some of the typical Ti-based alloys used in orthopedic and dental fields were subjected to acid and heat treatments with or without initial NaOH treatment, and their apatite formation in SBF was discussed in terms of their surface potential as well as surface structures and chemical compositions.

## 2. Results

### 2.1. Surface Structures

**Figure 1:**
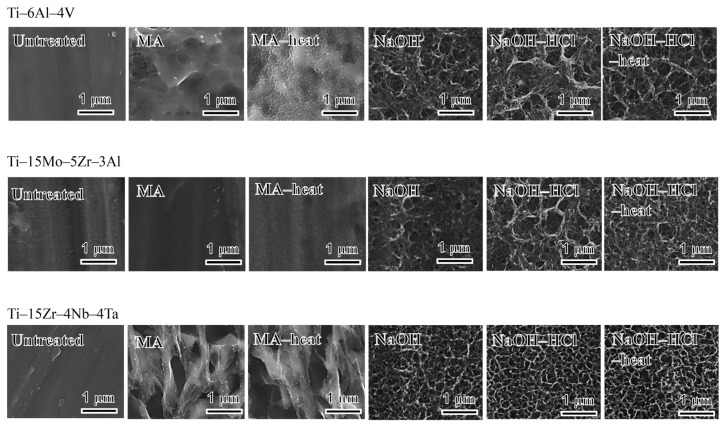
FE-SEM micrographs of the surfaces of various types of alloys untreated and subjected to mixed acid and subsequent heat treatments, or NaOH, HCl and subsequent heat treatments. MA: mixed acid treatment, MA‒heat: mixed acid and heat treatment, NaOH: NaOH treatment, NaOH‒HCl: NaOH and HCl treatment, NaOH‒HCl‒heat: NaOH, HCl and heat treatment.

Figure 1 shows field emission scanning electron microscope (FE-SEM) photographs of the surfaces of the alloy samples untreated and subjected to acid and heat treatments with or without the NaOH treatment. When the alloys were soaked in mixed acid solution, many corrodent pits in micrometer scale were formed on the surfaces of Ti–6Al–4V and Ti–15Zr–4Nb–4Ta, while no apparent changes were observed on the surface of the Ti–15Mo–5Zr–3Al. Subsequent heat treatment produced nano-sized spherical particles all over the surface of Ti–6Al–4V, while it caused no obvious changes on the surfaces of Ti–15Mo–5Zr–3Al and Ti–15Zr–4Nb–4Ta alloys. When the alloys were soaked in NaOH solution, network morphologies in nanometer scale were formed on the surfaces of alloys regardless the type of alloy. These morphologies were not changed even after the subsequent HCl and heat treatments.

Table 1, Table 2 and Table 3 show their surface chemical compositions measured by X-ray photoelectron spectroscopy (XPS) and energy dispersive X-ray spectrometry (EDX). It is shown in Table 1 that the relative ratios of alloying elements of Al and V to Ti in Ti–6Al–4V were essentially not changed by the mixed acid treatment, but increased markedly by the subsequent heat treatment. No apparent changes after the mixed acid treatment were observed on the chemical composition of the surface of Ti–15Mo–5Zr–3Al, as shown in Table 2. However, subsequent heat treatment decreased by half the ratios of Mo, Zr and Al to Ti. In contrast, it is shown in Table 3 that the ratios of the alloying elements of Zr, Nb, Ta and Pd to Ti were increased by the mixed acid treatment in the case of Ti–15Zr–4Nb–4Ta, but they were slightly decreased by the subsequent heat treatment. As a result, the ratio of Nb, Ta and Pd to Ti was doubled by the mixed acid and heat treatments. When the alloys were subjected to the NaOH treatment, the alloying elements were selectively removed from the alloy surfaces regardless of the type of the alloy while some amounts of sodium ions were incorporated. Subsequent HCl treatment completely removed the sodium ions. The ratios of the alloying elements to Ti remained low even after the HCl and the subsequent heat treatment.

**Figure 2:**
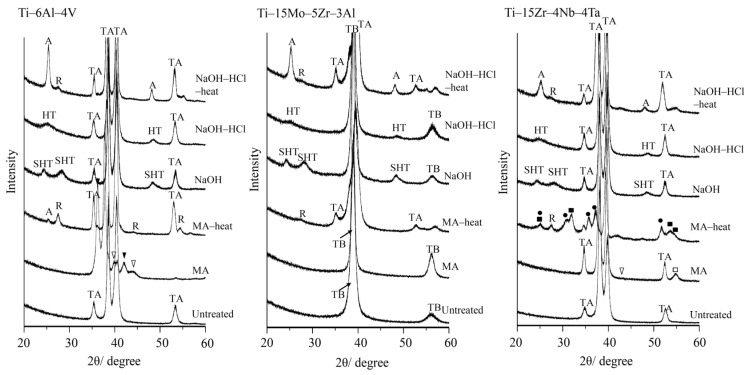
XRD profiles of the surfaces of Ti alloys untreated and subjected to mixed-acid and heat treatments or NaOH, HCl and heat treatments. TA: α-Ti, TB: β-Ti, A: Anatase. : TiH_0.71_, : TiH, : Nb_2_O_5_, : TiZrO_4_, R: Rutile.

Figure 2 shows thin-film X-ray diffraction (TF-XRD) profiles of the samples untreated and subjected to acid and heat treatments with or without the NaOH treatment. When the alloys were soaked in the mixed acid, titanium hydride, such as TiH (JCPDS file 00-040-1244) and TiH_0.71_ (JCPDS file 00-040-0980), were formed on the surfaces of Ti–6Al–4V, while no obvious changes were observed on the surfaces of Ti–15Mo–5Zr–3Al. Nb_6_O (JCPDS file 00-015-0258) accompanied by small amount of TiH were formed on the surfaces of Ti–15Zr–4Nb–4Ta. When these alloy samples were subsequently heat-treated, anatase and rutile type TiO_2_ were formed on Ti–6Al–4V while only a small amount of rutile was formed on Ti–15Mo–5Zr–3Al accompanied with a partial transformation from β- to α-Ti. In contrast, some alloy oxides such as Nb_2_O_5_ and TiZrO_4_ as well as rutile were formed on the Ti–15Zr–4Nb–4Ta surfaces. When these alloys were soaked in the NaOH solution, sodium hydrogen titanate, Na*_x_*H_2-*x*_Ti_3_O_7_ [28,34], was produced on the alloys regardless of the type of the alloy. This was transformed into hydrogen titanate, H_2_Ti_3_O_7_ [28,34], by the subsequent HCl treatment, and then into anatase and rutile by the final heat treatment regardless of the type of the alloy.

**Figure 3:**
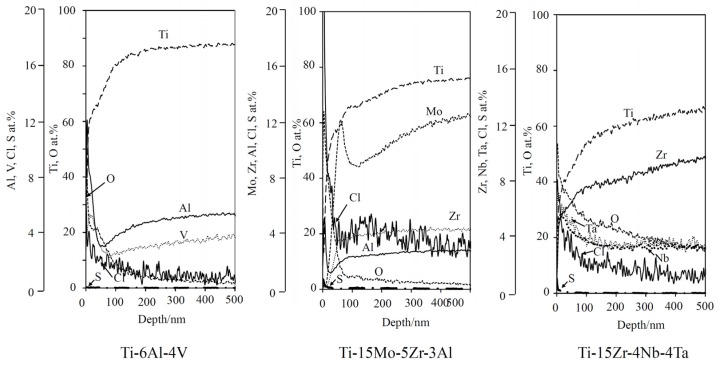
Glow discharge optical emission spectroscopy (GD-OES) depth profile of the surface of Ti alloys subjected to MA-heat treatment.

Glow discharge optical emission spectroscopy (GD-OES) depth analysis was performed on the surfaces of the alloys that were subjected to mixed acid and heat treatments to investigate the presence of acid roots of chloride and sulfate on the alloy surfaces, as shown in Figure 3. Some amount of chloride and sulfate were detected on the surfaces of alloys regardless of the type of alloy, indicating that these acid roots were adsorbed on the alloy surfaces during the mixed acid treatment and remained even after the heat treatment. It was found that oxygen penetration to the alloy substrate was less than 100 nm in thickness except Ti–15Zr–4Nb–4Ta alloy where it was more than 500 nm. It can be seen that Al and V in Ti–6Al–4V, Al in Ti–15Mo–5Zr–3Al, Nb and Ta in Ti–15Zr–4Nb–4Ta were enriched near the surfaces, whereas the other alloying elements were scarce. These distributions of the alloying elements were essentially consistent with the results of XPS and EDX analysis except Ti–15Mo–5Zr–3Al and Ti–15Zr–4Nb–4Ta where it was discovered that Ta and Al were enriched near the top surfaces of these alloys.

### 2.2. Zeta Potential

**Figure 4:**
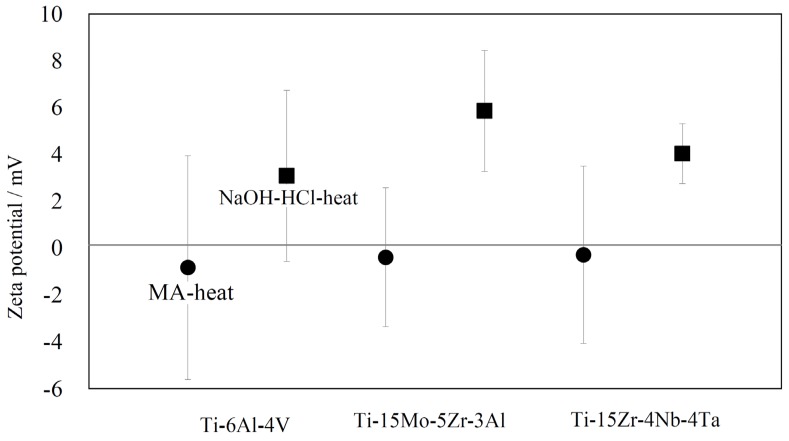
Zeta potential of Ti alloys subjected to MA-heat treatment (black circle) or NaOH-HCl-heat treatment (black square).

Figure 4 shows zeta potentials of the treated alloys. When the alloys were subjected to the mixed-acid and heat treatment, their zeta potentials were found to be almost zero (−0.8 mV for Ti–6Al–4V, −0.4 mV for Ti–15Mo–5Zr–3Al, −0.3 mV for Ti–15Zr–4Nb–4Ta) irrespective of the type of the alloy. In contrast, they became positively charged (3.1 mV for Ti–6Al–4V, 5.9 mV for Ti–15Mo–5Zr–3Al, 4.0 mV for Ti–15Zr–4Nb–4Ta) when they were initially subjected to the NaOH treatment followed by the HCl and heat treatments.

### 2.3. Apatite Formation

**Figure 5:**
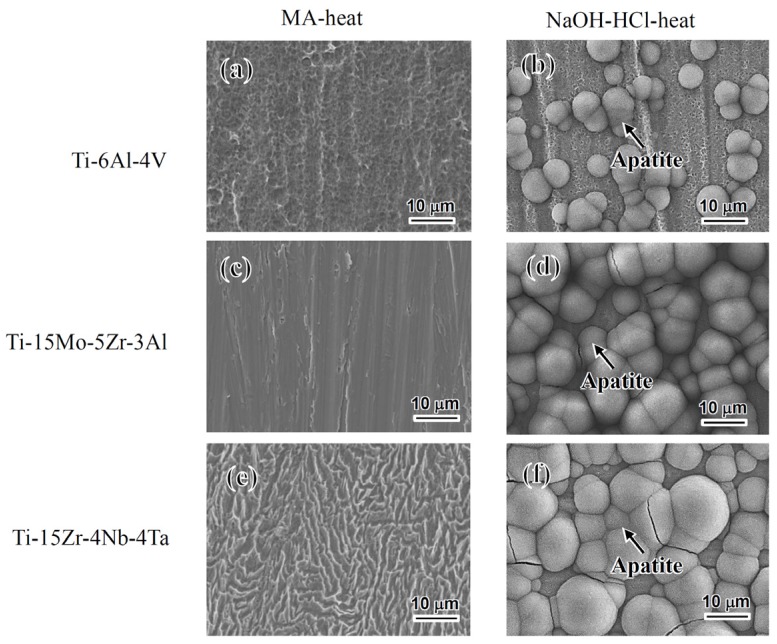
FE-SEM pictures of Ti alloys soaked in simulated body fluid (SBF) for 3 days following (**a**), (**c**), (**e**) MA-heat or (**b**), (**d**), (**f**) NaOH-HCl-heat treatment.

Figure 5 shows FE-SEM photographs of the surfaces of the alloys soaked in a simulated body fluid (SBF) for 3 days after the acid and heat treatments with or without the NaOH treatment. No precipitates were observed on the alloys subjected to the mixed-acid and heat treatment, as shown in Figure 5a,c,e. No or little precipitates were observed again even when the soaking period was prolonged up to 7 days. In contrast, many spherical precipitates were formed on the surfaces of the alloy subjected to NaOH-HCl-heat treatment regardless of the type of alloy within 3 days, as shown in Figure 5b,d,f. The amount of precipitates formed on Ti–15Mo–5Zr–3Al and Ti–15Zr–4Nb–4Ta were slightly larger than that on Ti–6Al–4V.

## 3. Discussion

Dynamic movements of alloying elements were developed by chemical and heat treatments, although they strongly depend on the kind of chemical treatment and type of alloy. This might be because of the different corrosion resistance of each element to acid or alkaline solution and affinity to oxygen at high temperature.

Mixed acid and heat treatments enriched the ratio of Al and V to Ti in Ti–6Al–4V, and the ratio of Nb, Ta and Pd to Ti in Ti–15Zr–4Nb–4Ta on the surfaces of the alloys, as shown in Table 1 and Table 3. In contrast, the ratio of Mo, Zr and Al to Ti in Ti–15Mo–5Zr–3Ta was decreased by the same treatment, although they still remain at least half, as seen in Table 2. According to XRD, some alloy oxides such as titanium zirconium oxide and niobium oxide were formed on the Ti–15Zr–4Nb–4Ta alloy, while only anatase and rutile of TiO_2_ were detected on the surfaces of Ti–6Al–4V, and Ti–15Mo–5Zr–3Ta alloys, as shown in Figure 2. This suggests that the alloying elements such as Al, V, Ta and Pd were replaced with Ti without apparent change in anatase and/or rutile structure. It was reported that various kinds of elements such as V, Nb and so forth were incorporated into the lattice structure of anatase [35,36,37]. The incorporation of a trace amount of these elements altered the electrical, photochemical, and magnetic properties of anatase [35,36,37].

When these alloys were soaked in SBF, none of them formed apatite within 3 days. This was in contrast with pure Ti subjected to the same treatments that formed large amounts of apatite particles within 1 day [4]. The surface structural changes and apatite formation process on pure Ti subjected to the same mixed acid and heat treatments were shown as follows [4].

When Ti was soaked in mixed acid solution of H_2_SO_4_ and HCl, acid roots of sulfate and chloride ions were adsorbed on its surface accompanied by the formation of a titanium hydride layer. These acid roots remained even after the subsequent heat treatment that resulted in the formation of a rutile titanium oxide layer on the metal surface. When the thus treated metal was soaked in SBF, it released the acid roots from the surface to form an acidic environment in the vicinity of the surface. As a result, the surface became positively charged, since titanium oxide is positively charged in an acidic environment [38]. The positively charged surface first selectively adsorbed the negatively charged phosphate ions on its surface. As the phosphate ions begin to accumulate, the surface becomes negatively charged, and, hence, combines with the positively charged calcium ions to form calcium phosphate. The calcium phosphate formed eventually transforms into stable crystalline apatite.

In the present study, acid roots of sulfate and chloride ions were detected on the alloy surfaces after the mixed acid and heat treatments regardless of the type of alloy, as shown in Figure 3. These acid roots are expected to be released to form an acidic environment in the vicinity of the surfaces, as is the case of pure Ti when the alloys are soaked in SBF. However, the zeta potentials of the alloys were almost zero in contrast with the case of Ti, as shown in Figure 4. This might be because of enriched/remained alloy elements such as Al, V, Nb, Ta, Zr, Mo and Pd on the surfaces. According to isoelectric points of Al_2_O_3_ (IEP_Al2O3_ = 9), V_2_O_5_ (IEP_V2O5_ = 2), Nb_2_O_5_ (IEP_Nb2O5_ = 4), Ta_2_O_5_ (IEP_Ta2O5_ = 5), ZrO_2_ (IEP_ZrO2_ = 7) and Mo_2_O_5_ (IEP_Mo2O5_ = 3) [39,40,41], surface layers of the alloys enriched with vanadium, niobium, tantalum and molybdenum oxides could decrease surface potential under physiological conditions. This could be the reason why the treated alloys did not form apatite as a result of suppression of the sequence adsorption of phosphate and calcium ions. It is reported that micro-scale roughness produced on pure Ti by acid treatment is effective in promoting osteoblast differentiation, although this roughness depresses osteoblast proliferation compared with a smooth surface [12,13,14,15]. Shi et al. reported that Ti–6Al–4V alloy subjected to heat treatment at 400 °C following mixed acid treatment of H_2_O_2_/HCl increased both differentiation and proliferation of MC3T3-E1 compared with the alloy subjected to the H_2_O_2_/HCl treatment alone [16]. These suggest that Ti–6Al–4V and Ti–15Zr–4Nb–4Ta alloys subjected to the MA-heat treatment in the present study may promote osteoblast differentiation and proliferation due to their micro-scale roughness. However, their bone bonding capacity would be limited because of a lack of apatite formation.

When the alloys were subjected to the NaOH treatment, alloying elements were selectively removed from the surfaces regardless of the type of alloy, as shown in Table 1, Table 2 and Table 3. Similar phenomena were reported on Ti–6Al–4V and Ti–15Zr–4Nb–4Ta in the literature [17,33]. Subsequent HCl and heat treatments essentially did not change the chemical compositions of the surfaces of the alloys, although some alloying elements were a little increased. As a result, only anatase and rutile were formed on the surface, irrespective of the kind of alloy. Thus, the treated alloys were shown to be positively charged, as seen in Figure 4, and formed apatite in SBF within 3 days, as shown in Figure 5b,d,f. It was reported that pure Ti with nano-porous sodium titanate produced by NaOH and heat treatment and the apatite-formed Ti generated by soaking the NaOH- and heat-treated metal in SBF for 2 days promoted osteoblast differentiation without suppression of proliferation compared with abraded Ti [26]. The bioactive Ti-based alloys prepared by NaOH-HCl-heat treatment in the present study may exhibit similar or higher promotion of osteoblast differentiation and proliferation since their surfaces are composed of nano-porous anatase and rutile, similar morphology to that of the NaOH- and heat-treated Ti and free from alkali ions. Because of their high capacity of apatite formation, it is expected that they would directly bond to living bone by forming a bone-like apatite layer on their surfaces [27].

It was shown that a porous Ti subjected to NaOH-HCl-heat treatment formed apatite in SBF and it was more deeply penetrated by newly grown bone compared with untreated Ti in rabbit femur [31]. The bone penetration was due to osteoconduction where the bone growth was induced by the surfaces of the pore walls. The porous Ti subjected to NaOH-HCl-heat treatment displayed not only osteoconduction, but also osteoinduction—ectopic bone formation in muscle [32]. This type of bioactive porous Ti was successfully subjected to clinical trials in the form of a spinal fusion device in five patients without autograft which is usually needed for conventional Ti spinal fusion devices [42]. It is expected that Ti-based alloys used in this study also exhibit osteoinduction as well as osteoconduction, and would thus be useful for spinal fusion devices. The porous Ti also has a beneficial effect in terms of mechanical compatibility with the surrounding bone since it exhibits a lower elastic modulus that is closer to that of human cortical bone compared with the solid body. This effect would be enhanced by replacing Ti with Ti-based alloys since higher porosity would be achieved due to the superior mechanical properties of the alloys; higher mechanical strength and compatible elastic modulus compared with Ti [43]. Furthermore, recent development of the additive manufacturing method such as a selective laser [44] or ion beam melting enable Ti and its alloys to be shaped in any complex including curved, graded and porous that would be perfectly fitted to the affected area of each patient. It is expected that a new type of custom-order Ti alloy device with high porosity and low elastic modulus for orthopedic and dental implants will be realized by combining the additive manufacturing method and bioactive treatments shown in this study.

## 4. Materials and Methods

### 4.1. Surface Treatments

Ti–6Al–4V, Ti–15Mo–5Zr–3Al and Ti–15Zr–4Nb–4Ta alloys with the chemical compositions shown in Table 4 were cut into rectangular-shaped samples 10 × 10 × 1 mm^3^ in size, abraded with #400 diamond plates, and ultrasonically cleaned with acetone, 2-propanol and ultrapure water for 30 min, and then dried at 40 °C overnight. These alloys were subjected to acid and heat treatment with or without NaOH treatment that are reported to be effective for pure Ti providing bone bonding as well as apatite formation [4,30]. For the simple acid and heat treatment (MA-heat treatment), the alloys were soaked in 5 mL of a mixture of 66.3% H_2_SO_4_ (*w/w*) solution and 10.6% HCl (*w/w*) solution in a weight ratio of 1:1 at 70 °C with shaking at a speed of 120 strokes/min for 1 h, then gently rinsed under the flow of ultrapure water for 30 s (MA treatment). Then, they were heated to 600 °C at a rate of 5 °C/min, kept at 600 °C for 1 h, followed by natural cooling in a Fe–Cr electrical furnace (heat treatment). For acid and heat treatment with initial NaOH treatment (NaOH-HCl-heat treatment), the alloys were soaked in 5 mL of 5 M NaOH solution at 60 °C, shaken at a speed of 120 strokes/min for 24 h (NaOH treatment) prior to an acid treatment in which the alloys were soaked in 10 mL of 50 mM HCl solution at 40 °C shaken at a speed of 120 strokes/min for 24 h (HCl treatment). In the NaOH-HCl-heat treatment, the dilute HCl was selected instead of the mixed acid in the MA-heat treatment since the surface layer formed by the initial NaOH treatment was completely dissolved when it was soaked in the mixed acid. The treated alloys were subjected to the heat treatment in the same manner as the heat treatment in MA-heat.

### 4.2. Surface Analysis

#### 4.2.1. Scanning Electron Microscopy

The surfaces of the metal samples untreated and subjected to the mixed acid and heat treatments were observed under a field emission scanning electron microscope (FE-SEM: S-4300, Hitachi Co., Tokyo, Japan) with a voltage of 15 kV.

#### 4.2.2. Energy Dispersive X-Ray Analysis and X-Ray Photoelectron Spectroscopy

The surface chemical compositions of the alloy samples untreated and subjected to the mixed acid and heat treatments were analyzed by an energy dispersive X-ray spectrometer (EDX: EMAX-7000, Horiba Ltd., Kyoto, Japan) at 5 kV for five areas, and their averaged value was used for analysis. The surface chemical composition of Ti–6Al–4V alloy was analyzed using X-ray photoelectron spectroscopy (XPS: ESCA-3300KM, Shimadzu Co., Kyoto, Japan) instead of EDX, since the peak positions of Ti and V were overlapped in EDX analysis. In XPS analysis, Mg-Kα radiation line was used as the X-ray source. The XPS take-off angle was set at 45 degrees. The binding energy of the measured spectra was calibrated by reference to the C1s peak of the surfactant CH_2_ groups on the substrate occurring at 284.6 eV.

#### 4.2.3. Thin-Film X-Ray Diffraction

The sample surfaces untreated and subjected to the mixed acid and heat treatments were analyzed using a thin-film X-ray diffractometer (TF-XRD: model RNT-2500, Rigaku Co., Tokyo, Japan), using a CuKa X-ray source at 50 kV and 200 mA. The glancing angle of the incident beam was set to an angle of 1° against the sample surface.

#### 4.2.4. Radio Frequency (RF) Glow Discharge Optical Emission Spectroscopy

The depth profiles of various elements on the surface of the samples subjected to the MA-heat treatment were analyzed using RF glow discharge optical emission spectroscopy (GD-OES, GD-Profiler 2, Horiba Co., Kyoto, Japan) under Ar sputtering at an Ar pressure of 600 Pa. RF electric field with power of 35 W was applied at a regular interval of 20 ms. The analysis was performed only on samples subjected to MA-heat treatment to investigate the presence of acid roots of chloride and sulfate because poor apatite formation was observed on the samples as shown in Figure 5a,c,e.

#### 4.2.5. Zeta Potential Measurement

Titanium alloy plates (size = 13 × 33 × 1 mm^3^) were prepared using the same method as described in Section 4.1, and these were subjected to the MA-heat or NaOH-HCl-heat treatment. The volume of the acid solution was increased to 15 mL in the mixed acid treatment and NaOH treatment while it was increased to 30 mL in the HCl treatment, according to the increased surface in this measurement. The treated alloy samples were grounded to allow for leakage of any stray charge, and they were immediately set in a zeta potential and particle size analyzer (Model ELS-Z1, Otsuka Electronics Co., Osaka, Japan) using a glass cell as the plate sample. The zeta potentials of the samples were measured under an applied voltage of 40 V in a 100 mM NaCl solution. The dispersant monitoring particles of polystyrene latex (size = 500 nm) were coated with hydroxyl propyl cellulose. Five samples were measured for each experimental condition and the average was used for analysis.

### 4.3. Soaking in a Simulated Body Fluid (SBF)

The samples subjected to the MA-heat or NaOH-HCl-heat treatments were soaked in 24 mL of a simulated body fluid (SBF) with various ion concentrations (Na^+^ 142.0, K^+^ 5.0, Ca^2+^ 2.5, Mg^2+^ 1.5, Cl^−^ 147.8, HCO_3_^−^ 4.2, HPO_4_^2−^ 1.0, and SO_4_^2−^ 0.5 mM) closed to human blood plasma at 36.5 °C. The SBF was prepared by dissolving reagent grade NaCl, NaHCO_3_, KCl, K_2_HPO_4_•3H_2_O, MgCl_2_•6H_2_O, CaCl_2_, and Na_2_SO_4_ (Nacalai Tesque Inc., Kyoto, Japan) in ultrapure water, and buffered at pH = 7.4 with tris(hydroxymethyl)aminomethane (CH_2_OH)_3_CNH_2_ and 1 M HCl (Nacalai Tesque Inc., Kyoto, Japan) at 36.5 °C [8]. After soaking in the SBF for 3 and 7 days, the samples were gently rinsed with ultrapure water, and dried.

## 5. Conclusions

The simple acid and heat treatment was not effective for inducing apatite formation on Ti-based alloys such as Ti–6Al–4V, Ti–15Mo–5Zr–3Al and Ti–15Zr–4Nb–4Ta, because the alloy surfaces were naturally charged even after the treatment due to enriched/remained alloying elements on their surfaces. In contrast, the acid and heat treatment with initial NaOH treatment was highly effective since the NaOH treatment selectively removes alloying elements from the alloy surfaces, resulted in positive surface charges. Thus, the treated alloys with a bioactive titania layer free from alkali ions would be useful as orthopedic and dental implants, especially in porous form, since they form apatite even in a living body and bond to living bone.

## Figures and Tables

**Table 1:** Results of X-ray photoelectron spectroscopy (XPS) quantitative analysis on the surfaces of Ti–6Al–4V alloy untreated and subjected to acid and heat treatments with or without NaOH treatment.

-	Element/at. %					Relative Ratio of M/Ti (M = Al, V)	
Treatment	O	Ti	Na	Al	V	Al/Ti	V/Ti
Untreated	66.1	28.9	0	4.7	0.3	0.161	0.009
MA	67.4	27.6	0	4.5	0.5	0.161	0.018
MA-heat	68.8	14.4	0	12.1	5.2	0.843	0.364
NaOH	58.8	23.1	17.6	0.5	0	0.022	0
NaOH-HCl	71.6	28.3	0	0	0	0	0
NaOH-HCl-heat	71.4	27.9	0	0	0.6	0	0.022

**Table 2:** Results of energy dispersive X-ray spectrometry (EDX) analysis on the surfaces of Ti–15Mo–5Zr–3Al alloy untreated and subjected to acid and heat treatments with or without NaOH treatment.

-	Element/at. %						Relative Ratio of M/Ti (M = Mo, Zr, Al)		
Treatment	O	Ti	Na	Mo	Zr	Al	Mo/Ti	Zr/Ti	Al/Ti
Untreated	7.1	80.9	0	6.5	2.4	3.2	0.080	0.030	0.040
MA	6.7	81.2	0	6.5	2.4	3.2	0.080	0.030	0.039
MA-heat	25.7	68.4	0	3.2	1.1	1.6	0.047	0.016	0.023
NaOH	61.9	31.5	6.6	0	0	0	0	0	0
NaOH-HCl	61.3	35.2	0	1.7	1.0	0.8	0.048	0.028	0.023
NaOH-HCl-heat	51.7	45.7	0	1.2	0.7	0.7	0.026	0.015	0.015

**Table 3:** Results of EDX analysis on the surfaces of Ti–15Zr–4Nb–4Ta alloy untreated and subjected to acid and heat treatments with or without NaOH treatment.

-	Element/at. %							Relative Ratio of M/Ti (M = Zr, Nb, Ta, Pd)			
Treatment	O	Ti	Na	Zr	Nb	Ta	Pd	Zr/Ti	Nb/Ti	Ta/Ti	Pd/Ti
Untreated	7.6	83.4	0	7.4	2.2	0.8	0.3	0.089	0.026	0.010	0.004
MA	28.6	53.7	0	8.0	6.0	2.9	0.8	0.149	0.112	0.054	0.015
MA-heat	54.7	38.6	0	3.3	2.1	0.9	0.4	0.085	0.054	0.023	0.010
NaOH	57.2	37.1	3.8	1.1	0.6	0.3	0.0	0.030	0.016	0.008	0
NaOH-HCl	52.6	44.6	0	1.3	1.3	0.6	0.1	0.029	0.029	0.013	0.002
NaOH-HCl-heat	56.3	42.6	0	1.8	1.0	0.4	0.1	0.042	0.023	0.009	0.002

**Table 4:** Chemical composition of Ti-based alloys.

Element/wt. %													
Alloy	Ti	Al	V	Nb	Ta	Mo	Zr	Pd	Fe	O	C	N	H
Ti–6Al–4V	Bal.	6.18	4.27	-	-	-	-	-	0.21	0.18	<0.002	0.005	0.002
Ti–15Mo–5Zr–3Al	Bal.	3.01	-	-	-	14.76	4.85	-	0.03	0.12	0.003	0.005	0.032
Ti–15Zr–4Nb–4Ta	Bal.	-	-	3.83	3.94	-	14.51	0.16	-	0.25	-	-	-
